# Soy-derived Phosphatidic Acid, Lysophosphatidic acid and Phosphatidylserine are sufficient to induce an increase in mTOR signaling

**DOI:** 10.1186/1550-2783-10-S1-P7

**Published:** 2013-12-06

**Authors:** David M Gundermann, Ralf Jäger, Martin Purpura, Troy A Hornberger

**Affiliations:** 1 Department of Comparative Biosciences, University of Wisconsin, Madison, WI, USA; 2 Increnovo LLC, 2138 E Lafayette Pl, Milwaukee, WI, USA

## Background

A protein kinase called the mechanistic target of rapamycin (mTOR) is a well-known regulator of cellular growth. In fact, several studies have indicated that the kinase activity of mTOR is required for mechanically-induced increases in skeletal muscle protein synthesis and hypertrophy. Previous studies have also determined that the lipid messenger phosphatidic acid (PA) plays a critical role in the stimulation of mTOR signaling and, an increase in PA concentration is sufficient for the activation of mTOR signaling. However, the mechanism by which PA stimulates mTOR is currently unknown. A primary target of mTOR includes the phosphorylation of p70 on the threonine 389 residue (P-p70-389), and thus, is a commonly accepted readout for the activation of mTOR. PA can be synthesized from a variety of reactions via multiple reactants. Therefore, the purpose of this study was to compare the effects of various PA precursors on their ability to stimulate mTOR signaling and determine if any other phospholipid species are also capable of stimulating mTOR signaling.

## Methods

C_2_C_12_ myoblasts were plated at approximately 30% confluence and grown for 24 hours in 10% FBS High Glucose DMEM. Cells were switched to 2mL/well serum free high glucose DMEM (no antibiotics) for 16 hours prior to the experiment. Cells were approximately 70% confluent at the time of the experiment. Cells were then stimulated for 20 minutes with vehicle (Control) or 10, 30 or 100µM of soy-derived phosphatidylserine (S-PS, SerinAid, Chemi Nutra, White Bear Lake, MN), phosphatidylinositol (S-PI), phosphatidylethanolamine (S-PE), phosphatidylcholine (S-PC), PA (S-PA, Mediator, Chemi Nutra, White Bear Lake, MN), lysophosphatidic acid (S-LPA), diacylglycerol (DAG), glycerol-3-phosphate (G3P), and egg-derived PA (E-PA). Cells were harvested in lysis buffer and subjected to immunoblotting. The ratio of P-p70-389 to total p70 was used as readout for mTOR signaling.

## Results

S-PI, S-PE, S-PC, DAG, and G3P elicited no increase in the ratio of P-p70-389 to total p70 compared to vehicle stimulated cells. In contrast, elevated mTOR signaling was observed at all tested concentrations of S-PS (529, 588, and 457%), S-LPA (649, 866, and 1,132%), and S-PA (679, 746, and 957%; P<0.05). Egg-PA induced an 873% increase in mTOR signaling with the 100µM dose (P<0.05), whereas no significant increase was observed with the 10 or 30µM doses.

## Conclusions

S-PA, S-LPA and S-PS are each sufficient to induce an increase in mTOR signaling. Therefore, they may be capable of enhancing the anabolic effects of resistance training and contributing to muscle accretion over time. Furthermore, S-PA is a more potent stimulator of mTOR signaling than PA derived from egg.

